# MALAT1 inhibits the Wnt/β-catenin signaling pathway in colon cancer cells and affects cell proliferation and apoptosis

**DOI:** 10.17305/bjbms.2019.4408

**Published:** 2020-08

**Authors:** Junjun Zhang, Qian Li, Bing Xue, Rui He

**Affiliations:** 1 Department of Pathology, Affiliated Hospital of Jining Medical University, Jining, Shandong, China; 2 Department of Pathology, Jining First People’s Hospital, Jining, Shandong, China; 3 Department of Union, West China Hospital, Sichuan University, Chengdu, Sichuan Province, China

**Keywords:** Colon cancer, MALAT1, proliferation, apoptosis, Wnt/β-catenin

## Abstract

Metastasis associated lung adenocarcinoma transcript 1 (MALAT1) is a highly conserved long noncoding RNA, which has been related to various pathological processes, including cancer. The role and mechanism of MALAT1 in colon cancer are not clear. We investigated MALAT1 expression in colon cancer tissues, the effect of MALAT1 on proliferation and apoptosis of SW480 cells, and the signaling pathway involved in the MALAT1 effects. MALAT1 expression was determined in 60 colon cancer and para-carcinoma tissues using reverse transcription polymerase chain reaction (RT-PCR). Knockdown of MALAT1 in SW480 cells was induced by small interfering RNA (siRNA), and the cells were divided into three groups: untreated control, nonsense siRNA-treated control, and MALAT1 siRNA-treated group. SW480 cell apoptosis was assessed using TUNEL assay and flow cytometry. Apoptosis-related and Wnt/β-catenin signaling pathway-related proteins were detected by Western blotting in SW480 cells. SW480 cell proliferation was assessed by CCK-8 assay. MALAT1 expression was significantly higher in colon cancer vs. para-carcinoma tissues. Knockdown of MALAT1 by siRNA increased the number of apoptotic cells and the apoptosis rate at 24 h post-transfection in SW480 cells. Bcl2 associated X protein (Bax) expression was increased, B-cell lymphoma 2 (Bcl-2) expression was decreased, and the ratio of cleaved caspase-3 to truncated caspase-3 was increased in MALAT1 siRNA-transfected SW480 cells. MALAT1 knockdown decreased the proliferation of SW480 cells at 24 h, 48 h, and 72 h post-transfection. Wnt and β-catenin expression was inhibited in MALAT1 siRNA-transfected SW480 cells. Inhibition of MALAT1 expression in colon cancer may promote apoptosis and hinder cell proliferation by suppressing the activation of Wnt/β-catenin signaling pathway.

## INTRODUCTION

Colon cancer is one of the most common malignant tumors of the digestive tract, characterized by high morbidity and mortality rates[1]. In Western countries, colon cancer is among the top five causes of mortality and the third major cause of cancer-related death [2]. Although the progression of colon cancer is a cumulative and complex process involving multiple factors, stages, molecular mechanisms, and genetic changes, the early development of colon cancer is slow and early diagnosis and treatment improve patient survival [3-5].

In mammals, the Wnt/β-catenin signaling pathway plays an important role in energy metabolism, cell proliferation and differentiation, growth, and development [6,7]. Abnormal activation of the Wnt/β-catenin pathway has been reported in colon cancer [8,9]. Therefore, targeting the Wnt/β-catenin pathway and its related components has a potential as a therapeutic approach in colon cancer.

Long noncoding RNAs (lncRNAa) are RNA transcripts of more than 200 nucleotides in length. Because lncRNAs are primarily involved in the regulation of gene transcription and posttranscriptional and epigenetic regulation, they have a significant role in the occurrence and development of various diseases [10].

Metastasis-associated lung adenocarcinoma transcript 1 (MALAT1) is nuclear-enriched lncRNA that has attracted great attention due to its regulatory function in cancer [11,12]. For example, the knockout of MALAT1 significantly inhibits the migration and proliferation of cervical cancer cells [13]. In bladder cancer cells, upregulation of MALAT1 activates the Wnt signaling pathway to promote the endothelial to mesenchymal transition of cancer cells, ultimately enhancing their metastatic capacity [14]. The posttranscriptional regulation of MALAT1 by miR-101 and miR-217 was reported to inhibit cell proliferation, migration, and invasion in esophageal squamous cell carcinoma [15]. However, the role and mechanism of MALAT1 in colon cancer are still poorly understood. In this study, we investigated MALAT1 expression in colon cancer tissues, the effect of MALAT1 on proliferation and apoptosis of SW480 cells, and the signaling pathway involved in the MALAT1 effects.

## MATERIALS AND METHODS

### Tissue specimens

A total of 60 surgical resection colon cancer and para-carcinoma specimens were obtained at the West China Hospital, Sichuan University from January 2017 to April 2018. All colon cancer cases were adenocarcinomas. After blood was washed with normal saline, the tissue specimens were cut into pieces, transferred into Eppendorf (EP) tubes, and stored in a refrigerator at -80°C.

### Ethics approval and consent to participate

The study was approved by the Ethics Committee of West China Hospital, Sichuan University and written informed consents were obtained from all patients or guardians.

### Materials

SW480 human colon cancer cell lines were purchased from the Biological Research Institute of the Chinese Academy of Sciences. Phosphate-buffered saline (PBS), trypsin, fetal bovine serum (FBS), and RPMI 1640 medium were purchased from Gibco (Rockville, MD, USA). Small interfering RNA (siRNA) was obtained from Google Biology (Wuhan, Hubei, China). Total RNA was extracted using TRIzol reagent (Invitrogen, Carlsbad, CA, USA). Complementary DNA (cDNA) was synthesized using the TaKaRa PrimeScript™ Kit (Takara Bio Inc., Otsu, Shiga, Japan).

### Cell culture

SW480 cells were cultured in an incubator with 5% CO_2_ at 37°C and digested with 0.25% trypsin-ethylenediaminetetraacetic acid (trypsin-EDTA). The cells were passaged when they completely covered the culture dish.

### MALAT1 knockdown

SW480 cells in the logarithmic growth phase were digested and inoculated into a 6-well plate. After 12 h (60–80% cells were fused), the complete medium was discarded. Cells were washed with serum-free medium for 2–3 times and starved in an incubator for synchronous growth. MALAT1 siRNA was dissolved in RNase-free deionized water to prepare transfection solution at a final concentration of 20 µmol/L. The cells were divided into three groups: untreated control, nonsense siRNA-treated control, and MALAT1 siRNA-treated group. The transfection solution was added into each well and fully mixed, followed by cell culture for 6 h. Then the solution was replaced with complete medium. The sequences of MALAT1 siRNA and nonsense siRNA are shown in Table 1.

**TABLE 1:**
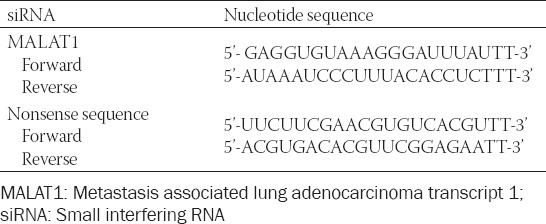
Sequences of MALAT1 siRNA and nonsense siRNA

### Reverse transcription polymerase chain reaction (RT-PCR)

The total RNA was extracted from SW480 cells and tissue specimens using the TRIzol reagent. The concentration and purity of RNA were detected using an ultraviolet spectrophotometer, and RNA at the concentration of 20 ng/µl and with the absorbance (A)_260_/A_280_ of 1.8–2.0 was used. mRNA was reverse transcribed into cDNA and stored in a refrigerator at -80°C. The PCR system included: 2.5 µL 10× Buffer, 2 µL cDNAs, 0.25 µL forward primers (20 µmol/L), 0.25 µL reverse primers (20 µmol/L), 0.5 µL deoxyribonucleoside triphosphates (10 mmol/L), 0.5 µL Taq enzymes (2×106 U/L), and 19 µL ddH_2_O. The PCR steps were as follows: 37°C for 1.5 h; 94°C for 5–10 min; and 60°C for 30 s for a total of 40 cycles. Glyceraldehyde 3-phosphate dehydrogenase (GAPDH) was used as the internal control. The primer sequences are shown in Table 2.

**TABLE 2:**
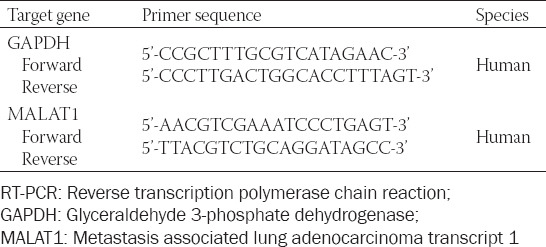
Primer sequences used in RT-PCR

### Western blotting

The culture medium was discarded and SW480 cells were washed with PBS three times. Lysis buffer (1000 µL) was added into each dish, which were then shaken for 20 min. The cells at the bottom of the dish were scraped off using a brush and placed into the EP tube. The collected cells were lysed using an ultrasonic pyrolyser for about 15 s. After standing for 15 min, the cells were centrifuged at 12000 rpm for 0.5 h. The supernatant was separated into EP tubes, and the protein concentration was determined by ultraviolet spectroscopy. All protein samples were adjusted to the same concentration (1 mg/ml). Protein samples were sub-packaged and placed in the refrigerator at -80°C. The total protein from SW480 cells was then analyzed by sodium dodecyl sulfate polyacrylamide gel electrophoresis (SDS-PAGE). The protein in the gel was transferred onto a polyvinylidene difluoride (PVDF) membrane, incubated with the primary antibody METTL16 (1:1000, #17676 from Cell Signaling Technology, Danvers, MA, USA) at 4°C overnight, and then incubated with a goat anti-rabbit secondary antibody (1:2000, #7074 from Cell Signaling Technology, Danvers, MA, USA) in a dark place for 1 h. The protein bands were scanned and quantified using an Odyssey scanner (LI-COR Biosciences, Lincoln, NE, USA), and the level of detected proteins was adjusted to GAPDH level.

### SW480 cell apoptosis by terminal deoxynucleotidyl transferase-mediated dUTP nick end labeling (TUNEL) assay

SW480 cells on slides were fixed with 4% paraformaldehyde solution in PBS for 1 h and washed with PBS three times. After permeabilization of cells, TUNEL reagent was added and two negative controls were set. After staining, the cells were observed, photographed, and counted under a fluorescence microscope.

### SW480 cell apoptosis by flow cytometry

SW480 cells in the logarithmic growth phase were digested with 0.25% trypsin-EDTA, prepared as a cell suspension, and inoculated into a 6-well plate. After centrifugation at 800 rpm for 5 min, precipitates were collected and washed twice with pre-cooled PBS. Then 75% ethanol was added and the solution was stored at 4°C for more than 4 h. The cells were centrifuged at 1500 rpm for 5 min and washed once with 3 mL PBS. Subsequently, 400 µL ethidium bromide (propidium iodide [PI], 50 µg/mL) and 100 µL RNase A (100 µg/mL) were added, and the cells were incubated at 4°C in the dark for 30 min. The apoptosis rate was detected according to the Annexin V-FITC PI apoptosis detection kit (Beyotime Institute of Biotechnology, Shanghai, China).

### SW480 cell proliferation by Cell Counting Kit-8 (CCK-8)

Cells were inoculated into 96-well plates with 4 000 cells/well, and 6 replicate wells were set in each group. A mixture of cholecystokinin-8 (GK10001, GlpBio, CA, USA) and culture medium (1:10) was added and placed in an incubator at 37°C for 2 h. The detection was carried out at 480 nm using a spectrophotometer (Mapada, Shanghai, China), representing optical density 1 (OD1). At 24, 48, and 72 h, the cell culture medium in well plates was washed with PBS twice. The mixture of CCK-8 and culture medium was added and placed in an incubator at 37°C for 2 h, followed by the detection at 480 nm using the spectrophotometer, representing OD2. Proliferation rate was calculated as (OD2 - OD1 - OD_empty_)/OD1 - OD_empty_.

### Statistical analysis

The experiments were repeated three times. IBM SPSS Statistics for Windows, Version 22.0. (IBM Corp., Armonk, NY, USA) was used for statistical analysis. Measurement data were expressed as mean ± standard deviation, and *t*-test was used for comparing data between two groups. A value of *p* < 0.05 was considered statistically significant.

## RESULTS

### Expression of MALAT1 in human colon cancer tissues and its correlation with clinicopathological features

**FIGURE 1:**
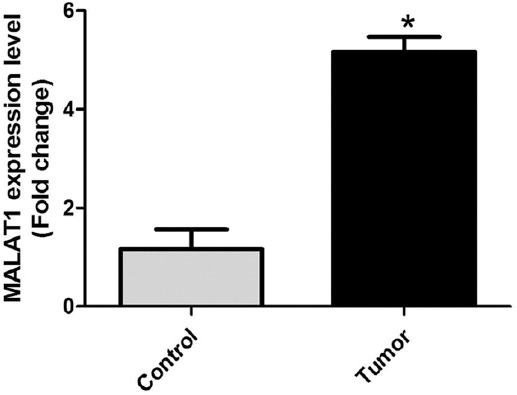
MALAT1 expression in human colon cancer tissues. Control: 60 para-carcinoma tissues; Tumor: 60 colon cancer tissues. MALAT1 expression in colon cancer tissues was more than 5 times higher than in para-carcinoma tissues (*p < 0.05). MALAT1: Metastasis associated lung adenocarcinoma transcript 1.

The RT-PCR analysis showed that MALAT1 expression in colon cancer tissues was significantly higher (i.e. more than 5 times) than in para-carcinoma tissues (*p* < 0.05; Figure 1). We further analyzed the association of MALAT1 expression with the clinicopathological features of colon cancer patients and showed no significant correlations with the age, gender, and degree of tissue differentiation (*p* > 0.05). The positive rate of MALAT1 expression was 91% in patients with metastasis and 67% in patients in the clinical stage III+IV (*p* < 0.05). The above results indicated that colon cancer patients with a low expression of MALAT1 may have a better prognosis (Table 3).

**TABLE 3:**
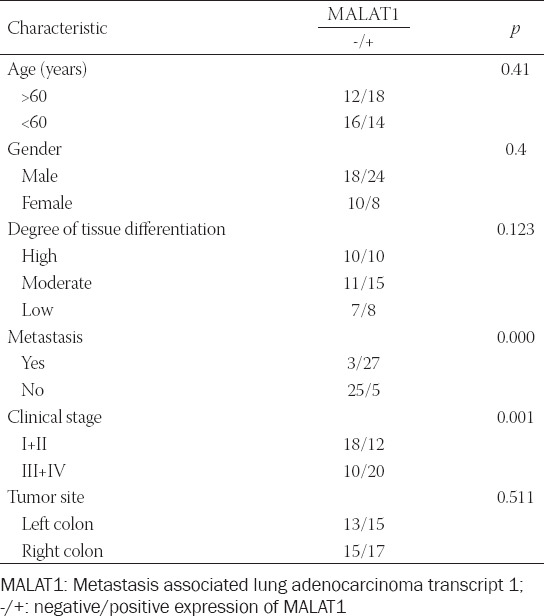
Correlation of MALAT1 expression with clinicopathological features of colon cancer patients

### Knockout of MALAT1 in SW480 cells

**FIGURE 2:**
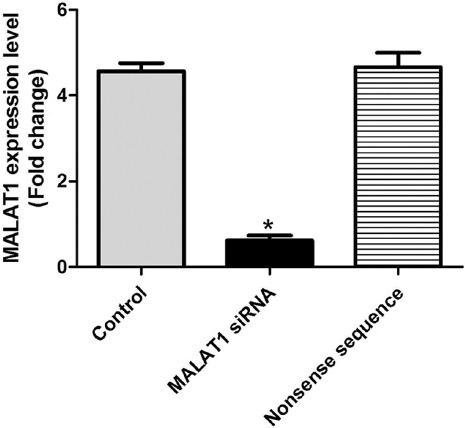
MALAT1 expression in SW480 colon cancer cell line transfected with MALAT1 siRNA. MALAT1 expression in MALAT1 siRNA group was significantly inhibited, it was about 20% of that in untreated control group (*p < 0.05). Control: Untreated control; MALAT1 siRNA: MALAT1 siRNA-treated group; Nonsense sequence: Nonsense siRNA-treated control; MALAT1: Metastasis associated lung adenocarcinoma transcript 1; siRNA: Small interfering RNA.

MALAT1 siRNA was transfected into SW480 cells, and the expression level of MALAT1 was detected by RT-PCR after 24 h. MALAT1 expression in MALAT1 siRNA group was significantly inhibited, about 20% of that in untreated control group (Figure 2), confirming that SW480 cell lines with MALAT1 knockout were successfully constructed.

### Effect of MALAT1 knockout on apoptosis-related proteins in SW480 cells

**FIGURE 3:**
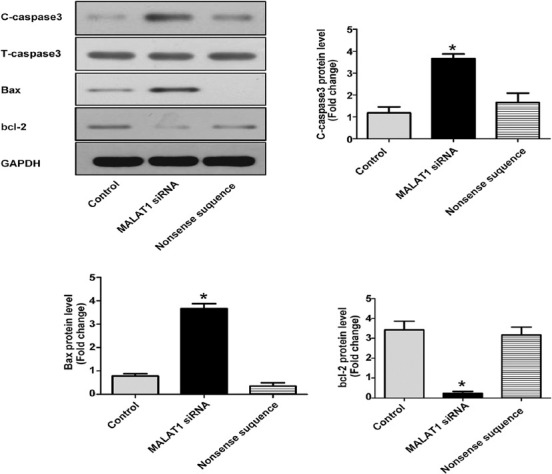
Effect of MALAT1 knockout on apoptosis-related proteins in SW480 colon cancer cells. At 24 h after MALAT1 knockout, the ratio of cleaved caspase-3 to truncated caspase-3 and the level of pro-apoptotic Bax were increased in MALAT1 siRNA group, while the level of anti-apoptotic Bcl-2 was significantly decreased (*p < 0.05 vs. untreated control group). Control: Untreated control; MALAT1 siRNA: MALAT1 siRNA-treated group; Nonsense sequence: Nonsense siRNA-treated control; MALAT1: Metastasis associated lung adenocarcinoma transcript 1; siRNA: Small interfering RNA; C: Cleaved; T: Truncated; Bcl-2: B-cell lymphoma 2; Bax: Bcl2 associated X protein.

At 24 h after MALAT1 knockout in SW480 cells, apoptosis-related proteins were detected by Western blotting. In MALAT1 siRNA group, the ratio of cleaved caspase-3 to truncated caspase-3 and the level of pro-apoptotic Bcl2 associated X protein (Bax) were obviously increased (*p* < 0.05 vs. untreated control), while the level of anti-apoptotic B-cell lymphoma 2 (Bcl-2) was significantly decreased (*p* < 0.05 vs. untreated control; Figure 3), indicating that the knockout of MALAT1 promotes apoptosis in colon cancer cells.

### Effect of MALAT1 knockout on apoptosis of SW480 cells by TUNEL assay

**FIGURE 4:**
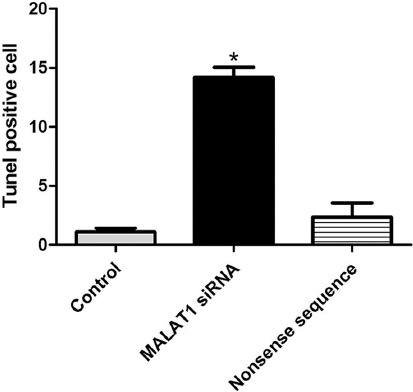
The number of apoptotic cells in SW480 colon cancer cell line transfected with MALAT1 siRNA, determined by TUNEL assay. The number of apoptotic cancer cells in MALAT1 siRNA group was about 15 times higher than in untreated control group (*p < 0.05). Control: Untreated control; MALAT1 siRNA: MALAT1 siRNA-treated group; Nonsense sequence: Nonsense siRNA-treated control; MALAT1: Metastasis associated lung adenocarcinoma transcript 1; siRNA: Small interfering RNA.

To further explore the effect of MALAT1 knockout on the apoptosis of SW480 cells, TUNEL staining was performed to quantify the number of apoptotic colon cancer cells. The number of apoptotic cancer cells in MALAT1 siRNA group was about 15 times higher than in untreated control group (*p* < 0.05; Figure 4), suggesting again the pro-apoptotic effect of MALAT1 knockout on colon cancer cells.

### Effect of MALAT1 knockout on apoptosis of SW480 cells by flow cytometry

**FIGURE 5:**
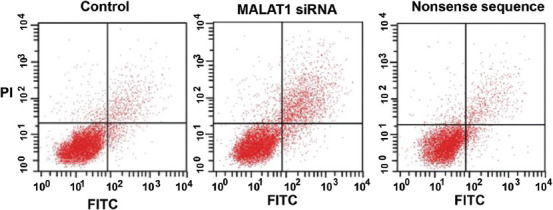
Apoptosis rate in SW480 colon cancer cell line transfected with MALAT1 siRNA, detected by flow cytometry. The apoptosis rate was the highest in MALAT1 siRNA group. Control: Untreated control; MALAT1 siRNA: MALAT1 siRNA-treated group; Nonsense sequence: Nonsense siRNA-treated control; MALAT1: Metastasis associated lung adenocarcinoma transcript 1; siRNA: Small interfering RNA; PI: Propidium iodide; FITC: Fluorescein isothiocyanate.

The apoptosis of SW480 cells at 24 h after MALAT1 knockout was also detected by flow cytometry. The apoptosis rate in untreated control, MALAT1 siRNA, and nonsense siRNA group was 5.82% ± 1.23, 24.51% ± 2.31, and 5.22% ± 1.46, respectively (*p* < 0.05; Figure 5). It is obvious that the apoptosis rate was the highest in MALAT1 siRNA group, confirming the promoting effect of MALAT1 knockout on apoptosis in colon cancer cells.

### Effect of MALAT1 knockout on proliferation of SW480 cells by CCK-8 assay

**FIGURE 6:**
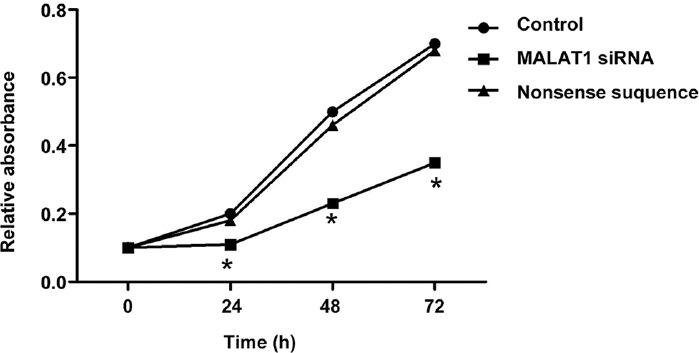
Cell proliferation in SW480 colon cancer cell line transfected with MALAT1 siRNA, detected by CCK-8 assay. MALAT1 knockout had an inhibitory effect on the proliferation of colon cancer cells, with a statistically significant difference compared to untreated control group at 24 h, 48 h, and 72 h (*p < 0.05). Control: Untreated control; MALAT1 siRNA: MALAT1 siRNA-treated group; Nonsense sequence: Nonsense siRNA-treated control; MALAT1: Metastasis associated lung adenocarcinoma transcript 1; siRNA: Small interfering RNA.

We further investigated the effect of MALAT1 knockout on the proliferative activity of SW480 cells by CCK-8 assay. As shown in Figure 6, MALAT1 knockout had an inhibitory effect on the proliferation of colon cancer cells, with a statistically significant difference compared to untreated control group at 24 h, 48 h, and 72 h (*p* < 0.05). All the above results suggest that the knockout of MALAT1 in colon cancer cells inhibits proliferation and promotes apoptosis.

### Effect of MALAT1 knockout on the Wnt/β-catenin signaling pathway

**FIGURE 7:**
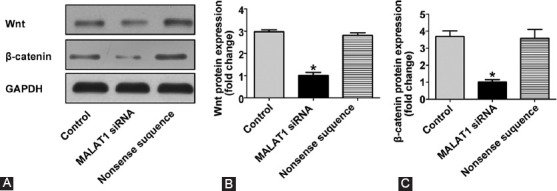
Effect of MALAT1 on the Wnt/β-catenin signaling pathway in SW480 colon cancer cell line transfected with MALAT1 siRNA. (A) Western blotting analysis of Wnt and β-catenin protein expression; (B) quantification analysis of Wnt protein expression; (C) quantification analysis of β-catenin protein expression. The expression of Wnt and β-catenin was significantly decreased after MALAT1 knockout in SW480 cells (*p < 0.05 vs. untreated control group). Control: Untreated control; MALAT1 siRNA: MALAT1 siRNA-treated group; Nonsense sequence: Nonsense siRNA-treated control; MALAT1: Metastasis associated lung adenocarcinoma transcript 1; siRNA: Small interfering RNA.

Considering the important role of the Wnt/β-catenin pathway in the occurrence and development of colon cancer we investigated whether the activation of the Wnt/β-catenin pathway in colon cancer is regulated by MALAT1. Wnt and β-catenin protein expression was quantified by Western blotting in untreated control, nonsense siRNA-treated control, and MALAT1 siRNA-treated SW480 cells. As shown in Figure 7, the expression of Wnt and β-catenin was significantly decreased after MALAT1 knockout in SW480 cells (*p* < 0.05 vs. untreated control), suggesting that the regulatory effects of MALAT1 on the proliferation and apoptosis of colon cancer cells occurred through the inhibition of the Wnt/β-catenin signaling pathway.

## DISCUSSION

Recently, the mortality rate of colon cancer has been increasing year by year. The occurrence and development of colon cancer are affected by genetic changes and, with the development of molecular biology and bioinformatics techniques, more oncogenes and tumor suppressor genes have been associated with colon cancer. Although the clinical symptoms and diagnostic criteria for colon cancer are relatively clear and standardized, the mechanisms underlying the progression of colon cancer cells, including proliferation, migration, apoptosis, and angiogenesis, remain poorly understood. In this study, we found that the knockout of MALAT1 lncRNA inhibited the proliferation and promoted the apoptosis of colon cancer cells and that both effects may be related to the inhibition of the Wnt/β-catenin pathway by MALAT1 knockout.

The role of lncRNAs in health and disease has attracted great attention in recent years. LncRNAs regulate different genes in the human body through transcriptional, post-transcriptional, and epigenetic regulation, thus affecting major biological processes in the organism. In particular, lncRNAs can exert tumor-inhibiting or tumor-promoting effects in various tumors. In the case of MALAT1 lncRNA, transforming growth factor beta (TGF-β) can promote the epithelial to mesenchymal transition in bladder cancer cells by upregulating MALAT1 [16]. In addition, MALAT1 can enhance the expression of genes related to cell proliferation, such as *MYBL2*, by inhibiting p53 activation, ultimately leading to increased cell proliferation in testicular ischemia-reperfusion injury[17].

Cell proliferation and apoptosis have an important role in the occurrence and development of cancer. Previous research has shown that the Wnt/β-catenin signaling pathway is involved in cell proliferation and apoptosis in various tumors, and it may have a completely opposite regulatory effect on apoptosis in different disease models. For example, the *BRAF* V600E mutation inhibits the activation of the Wnt/β-catenin pathway, promoting the apoptosis of melanoma cells. Therefore, a variety of BRAF (V600E) inhibitors can be clinically used as promising drugs in the treatment of melanoma [18,19]. In mouse liver model, the Wnt/β-catenin pathway inhibits the activation of forkhead box O3 (FOXO3) transcription factor to suppress apoptosis caused by oxidative stress [20]. However, in hematopoietic progenitor cells, the activation of the Wnt/β-catenin signaling pathway induces the apoptosis mediated by mitochondria [21]. The results of our study showed that the knockout of MALAT1 promoted apoptosis through the inhibition of the Wnt/β-catenin pathway, though such an effect of MALAT1 may be related to the different apoptotic pathways.

A number of studies have shown the specific role of the Wnt/β-catenin pathway in the proliferation of colon cancer cells. For example, the traditional Chinese medicine berberine inhibits the proliferation of colon cancer cells by inactivating the Wnt/β-catenin pathway [22]. Aesculetin exerts a similar effect through a direct targeted inhibition of β-catenin [23].

The results of our study suggest that MALAT1 acts as an endogenous inhibitor of Wnt/β-catenin in colon cancer cells, affecting cell proliferation and apoptosis. Another study showed that compounds such as resveratrol regulate MALAT1 expression in colorectal cancer (CRC) cells and inhibit tumor metastasis via the Wnt/β-catenin pathway [24]. Furthermore, Xu et al. demonstrated that the 6918 nt-8441 nt fragment located at the 3’ end of MALAT-1 plays an important role in cell proliferation, migration, and invasion in SW480 cells [25]. In the study of Ji et al. [26], the overexpression of MALAT1 promoted CRC cell proliferation and migration *in vitro* and tumor growth and metastasis in nude mice. They also suggested the mechanism underlying the MALAT1 effects, which involved the tumor suppressor gene *SFPQ* and proto-oncogene *PTBP2*. Finally, MALAT1 and polypyrimidine tract binding protein 2 (PTBP2) were overexpressed in CRC tissues and associated with the invasion and metastasis of CRC in their study [26]. Other studies showed that an increased MALAT1 expression promotes the proliferation of colon cancer cells [27,28], although by regulating other targets such as *SOX9* and miR-129-5p/HMGB1 axis.

The following are the limitations of our study: 1) our results were not verified by cell experiments; 2) only one type of colon cancer cell line was used; 3) the direct target of MALAT1 in colon cancer cells was not investigated; 4) the initiation of apoptosis involves different pathways, and it is not clear whether the initiation of apoptosis in colon cancer cells induced by MALAT1 knockdown is dependent on the extrinsic pathways (i.e. Fas and tumor necrosis factor pathways) or the intrinsic mitochondrial pathway. The above questions remain to be explored. Despite these limitations, our study revealed that MALAT1 exerts an important regulatory effect on colon cancer cells.

## CONCLUSION

Inhibition of MALAT1 can promote the apoptosis and hinder the proliferation of colon cancer cells by restraining the activation of the Wnt/β-catenin signaling pathway, ultimately exerting antitumor effects. MALAT1 is expected to be a new target in the treatment of colon cancer in the future.
